# Primary headache disorders in adolescents in North- and South-Tyrol: Findings of the EVA-Tyrol-Study

**DOI:** 10.1177/03331024221088997

**Published:** 2022-03-25

**Authors:** Katharina Kaltseis, Florian Frank, Benoît Bernar, Sophia Kiechl, Bernhard Winder, Ursula Kiechl-Kohlendorfer, Michael Knoflach, Gregor Broessner, for the Early Vascular Ageing (EVA) Tyrol Study Group

**Affiliations:** 1 Department of Neurology, Medical University of Innsbruck, Innsbruck, Austria; 2 Department of Paediatrics I, Medical University of Innsbruck, Innsbruck, Austria; 3 VASCage, Research Centre on Vascular Ageing and Stroke, Innsbruck, Austria; 4 Department of Paediatrics II, Medical University of Innsbruck, Innsbruck, Austria

**Keywords:** Migraine, adolescents, headache, preventive medication, life-style modification

## Abstract

**Objective:**

Assessment of the prevalence of primary headache disorders, associated risk factors and use of acute/preventive medication in a representative large sample of adolescents.

**Methods:**

Within the EVA-Tyrol project, a community-based non-randomized controlled cross-sectional study, data was collected from adolescents aged 14–19 years from 45 sites across North-, East- and South Tyrol. Headaches were classified according to the latest ICHD-3 and assessed by headache specialists in face-to-face interviews.

**Findings:**

Of 1923 participants 930 (48.4%) reported having headaches. Female to male ratio was 2:1. Migraine, tension-type headache and other headache were diagnosed in 10%, 30.2% and 8.2% respectively. Medication overuse was diagnosed in 3.4%, increasing up to 21.7% in participants with chronic headache. The use of preventative medication was not reported by any adolescent. Sleep disturbances (p < 0.05), alcohol consumption (p < 0.05), low physical activity (p < 0.01) and high screen time exposure (p < 0.01) were associated with an increased risk of headaches.

**Conclusion:**

We report high prevalence of primary headache disorders and medication overuse in a large community-based sample of teenagers. Acute and preventive non-drug and pharmacological treatments are not established due to lack of paediatric headache outpatient clinics. Promoting health education in teenagers and encouraging public awareness, including that of health care providers is pivotal.

**Trial registration:** EVA-Tyrol has been retrospectively registered at clinicaltrials.gov under https://clinicaltrials.gov/ct2/show/NCT03929692 since April 29, 2019.

## Introduction

Headache disorders are a widespread neurological condition in adolescents, but still underdiagnosed und undertreated. A systematic review of the available data on headache in children and adolescents estimated the overall prevalence at 58.4% (1). In the young population, primary headache disorders, thus headaches without underlying cause, predominate. Of these, migraine and tension-type headaches (TTH) are the most common whereas trigemino-autonomic cephalalgias are rare among teenagers (2). The estimated prevalence of migraine and tension type headache in school-aged children varies widely on account of age, data collection and headache classification used. Migraine is estimated to affect around 10% (1,3) of teenagers whereas the prevalence of TTH is between 10-25% (4,5). Yet most of these reviews are outdated and have not been done using the latest version of the ICHD classification published in 2018 (6).

Several risk factors for the development of primary headache disorders in adolescents have been discussed, and include irregular sleeping patterns, dehydration, irregular meals, physical inactivity, and high screen time exposure (7–9). Lifestyle of young people has always been challenging and changing rapidly. But within the last 10 to 15 years, with the rocket rise of social media and the consecutive influence on media consumption, leisure behaviour and peer group pressure, a possible influence on primary headache could be assumed. Therefore, community-based face-to-face interviews using up-to-date classification is of utmost interest to the headache community.

Whereas significant clinical progress has been achieved in the therapy of adult migraine patients, pharmacological interventions in adolescents remain poorly studied. Guidelines on the use of acute medication in headache in children recommend the use of ibuprofen and acetaminophen as well as triptans if the migraine headache attacks do not respond adequately to NSAIDs or other analgesics (10). Little is known about the current use of acute or preventative treatment patterns in adolescents in general practice. However, a survey of adult Austrian migraine patients was published in 2018 and less than 6% used triptans as acute medication (11).

The aim of the present study was to assess the prevalence of primary headache disorders, associated risk factors as well as to collect information on the use of acute/preventative medication in a large community-based sample of adolescents. Herein, we used a cross-sectional design with recruitment of adolescents from all possible education settings including high schools, profession-oriented schools, and apprentices in training companies – including a structured face-to-face interview with headache specialists.

## Methods

### Study Population

Study participants were originally recruited as part of the Early-Vascular-Ageing (EVA)- Tyrol-Study (12), a community-based non-randomized controlled cross-sectional study, which was conducted between May 2015 and July 2018 at 45 institutions in East- and North-Tyrol (Austria) and Bruneck (South-Tyrol, Italy). The aim of the study was to assess and promote the cardiovascular health of an unselected cohort of healthy adolescents aged 14-19 years.

In brief, data was collected by a paper case report form (CRF) and included self-administered and assisted questionnaires, a structured headache interview, and a series of examinations (blood sampling, high-resolution ultrasound of the carotid arteries, tonometric measurement of carotid-femoral pulse-wave velocity, blood-pressure measurement, and anthropometry). Data acquisition was performed by full time neurologists (especially trained in headache medicine), paediatricians, medical doctors and assisted by medical students. Examinations and interviews were performed on site at participating schools or training companies.

**Figure 1.**
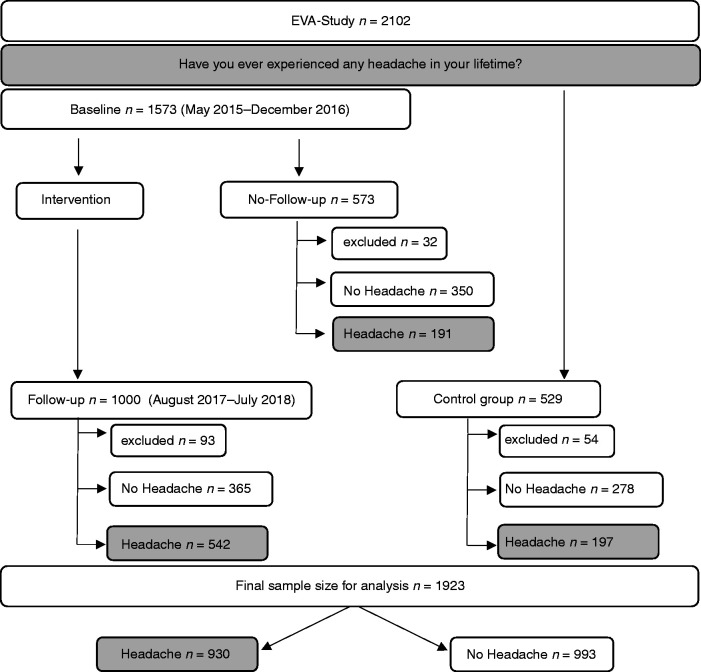
Study Flow Chart. 2102 adolescents were included in the EVA-Tyrol study and all of them were amongst other things asked, “Have you ever experienced any headache in your lifetime?” Of the randomly selected 1573 adolescents that completed the baseline examination and received cardiovascular health counselling, 1000 participated in the follow-up examination after approximately 2 years. 529 adolescents without participation in a health promotion program served as a control group. 179 adolescents were excluded from the analysis due to missing data and/or age >19 years at examination. Of the final sample of 1923 teenagers, 930 experienced headaches and 993 did not report any.

From the 2102 adolescents who participated in the study 1573 were randomly selected to an intervention group and received individual counselling in cardiovascular risk behaviour and risk factors. Of those, 1000 participated in a follow-up examination within two years (mean 22.1 ± 3.1 months). Participants were attending 9th and 10th grade of high school and profession-oriented school or apprentices in training companies (mean age, 14–16 years) at baseline and 12th grade or apprentices (mean age, 17–18 years) at the follow-up examination. 529 students, with a mean age of 17–18 years, were recruited as a control group and did not receive any interventions. A detailed description of the methods has been published before (12–14). For the current analysis data from the follow-up examination (n = 1000), the baseline examination of those who did not participate in the follow-up (n = 573) and the control group (n = 529) was used (Figure 1).

All participants provided written informed consent and minors provided assent along with the permission of their parents or legal guardian. The study protocol was approved by the local ethics committee of the Medical University of Innsbruck (AN 2015-0005 345/4.13) and was executed in agreement with the Declaration of Helsinki. The trial has been registered at clinicaltrials.gov (NCT03929692).

### Headache Type

Characteristics of headaches and information about the use and the category of acute medication were collected by trained headache specialists in a structured face-to-face interview. Headaches were classified according to the latest ICHD-3 (6). Participants were asked if they have ever experienced headache in their lifetime. Adolescents not reporting any headaches were allocated to the “no headache” group. Participants answering affirmatively, were interviewed about the headache characteristics in detail. Information on frequency, location, quality, aggravation by routine physical activity, duration, intensity, and accompanying symptoms was collected during the interview. Furthermore, they were asked if they experienced visual, sensory, or motor symptoms at any time before/during the headache attack or in isolation. According to their description, the headache specialists categorized the participants either in the “Migraine” (with and without aura, including probable migraine) or “Tension-type-Headache” (including probable TTH) group. Headaches that could not be assigned to either of the 2 entities were included in the “other headache” group. Information on the frequency of headache days within in the last 3 months before the interview was collected. <1 headache day/month was classified as “infrequent headache”, >1 and <15 headache days/month was rated as “episodic headache” and 15 or more headache days/month were categorized as “chronic headache”. On-demand medication categories included none, NSAID single agent preparation, NSAID combination preparation, Triptan, Ergotamine, Opioids and other. In accordance with the ICHD-3-guidelines, medication overuse was defined as the use of one or more non-opioid analgesics (NSAIDs, paracetamol or acetylsalicylic acid) on 15 or more days/month or the use of triptans, ergotamines, opioids or combination analgesics on 10 or more days/month for headache treatment.

### Sociodemographic data

Information on APGAR (Appearance, Pulse, Grimace, Activity, and Respiration) Score, mode of birth (spontaneous, vacuum extraction, caesarean) and information on breastfeeding were derived from hospital records or a mother-child booklet. Both the Austrian and the Italian mother-child booklets are official European health records covering pregnancy, birth, and the first 5 years of life, during which all Austrian and Italian children attend regular examinations in paediatric practices.

Health behaviours and life-style factors as well as information on life satisfaction were assessed by a case report form adapted from the Health Behaviour in School-aged Children Survey Study, Bruneck Study and the Atherosclerotic Risk Factors in Male and Female Youngsters Study (15–18). The Family Affluence Scale III was used to assess the socio-economic status (19). According to the answers, the socio-economic status was categorised into low (0–3 points), medium (4–6 points), and high (7–9 points). To assess physical activity, days/week with at least 60min of exercise were collected.

Alcohol consumption was assessed in a dichotomous manner (yes/no) during an in-person interview. If adolescents answered affirmatively, they were asked to disclose the type, amount, and frequency of the consumed beverages. Subsequently, the alcohol intake per week was calculated using the formula: $\mathrm{amount}\;of\;\mathrm{alcohol}\mathrm{beverage}\;in\;\mathrm{milliliters}\times \frac{\mathrm{Vol}\%}{100}\times 0.8=\mathrm{alcohol}in\;\mathrm{grams}.$

Adolescents were categorized as smokers if they ever smoked a whole cigarette.

Family history was obtained regarding cardiovascular disease (CVD) (premature CVDs (women <65 years, men <55 years), hypertension in one 1st or two 2nd degree relatives) but not for headache disorders.

Physical examination included weight and height. Current weight was assessed using medical precision scales. The current height was determined using a Harpenden stadiometer (Holtain, Crymich, United Kingdom). BMI was computed as follows: BMI = kg/m^2^.

### Statistical Methods

Descriptive statistics was used for characterization of the study population according to their headache diagnosis. Prevalence estimates (%) for any headache and for each headache type were calculated for the total sample and for each gender. Total numeric values are presented as mean ± standard deviation (SD).

We used multinomial logistic regression models to calculate odds ratios (ORs) and 95% confidence intervals (CIs). We ran multivariable models adjusting for: gender (female, male), age (<16, 16, 17, 18), body-mass-index (BMI, 4 categories: <18.5 kg/m^2^; 18.5–24.9 kg/m^2^; 25–29.9 kg/m^2^; ≥30 kg/m^2^), screen time exposure (clustered from less than 0.5 hours/day up to more than 7 hours/day), smoking (yes/no), alcohol consumption (yes/no), sleep disturbance (yes/no), school type (high school, profession-oriented school and apprentices) and regular exercise (according to the WHO guidelines for children aged 5–17 years 3 days/week with at least 60 minutes of sport (20)). The “No-headache” category was used as a reference group. Variables were chosen based on the literature on probable risk factors for primary headache disorders in adolescents.

Between group differences, we performed Student’s t-test for parametric variables and Kruskal Wallis for nonparametric variables. We tested differences according to sociodemographic characteristics using chi-squared-test. Missing data was addressed by listwise exclusion from further analysis.

We considered a p-value <0.05 to be statistically significant. All analyses were performed using statistical software (SPSS version 26.0; IBM Corporation, Armonk, NY, US).

## Results

### Sample characteristics

In the current study, 2102 participants were recruited. 179 adolescents were excluded for this respective analysis due to an age of 19 years or older or because of missing headache data. The final sample size consisted of 1923 adolescents, 821 (42.7%) were male and 1102 (57.3%) were female. Mean age was 17.0 ± 1.0 years. 618 (31.2%) of the participants attended high-schools, 1054 (54.8%) were from a profession-oriented school and 251 (13.1%) were apprentices.

### Headache prevalence

Of the 1923 participants, 993 (51.6%) reported not having a headache. 193 (10.0%) were diagnosed with migraine, 580 (30.2%) with tension-type headache and 157 (8.2%) with other headache. 51 (2.6%) of the participants who were diagnosed with migraine reported aura symptoms but due to the small sample size migraine with and without aura were combined in the analysis. Most of the participants (607; 65.5%) reported having episodic headache (i.e. >1 and <15 headache days/month), 211 (22.8%) reported having infrequent headaches (i.e. <1 headache day/month) and 109 (11.9%) reported chronic headaches (i.e. ≥15 headache days/month). The mean headache days/month were 3.5 ± 7.1 days. Mean headache days/month in adolescents suffering from chronic headache was 22.4 ± 6.4 days/month but 41 (37.6%) reported having daily headaches. Of those reporting chronic headaches, migraine, TTH and other headache was diagnosed in 20.2%, 56.0% and 23.9% respectively.

Table 1 summarizes the association between characteristics of the study population and headache type. We could not find a possible confounder (i.e. sex and school type) explaining the observed decrease in all headache types in the age group of >18. None of the participants reported headache indicative of other primary headache syndromes.

**Table 1.** Characteristics of the study population according to the headache types.

* *	Headache Types							
	Migraine		TTH		Other headache		No headache	
	(n = 193)		(n = 580)		(n = 157)		(n = 993)	
	n	%	n	%	n	%	n	%
Gender
Male	76	39.4	187	32.2	46	29.3	512	51.6
Female	117	60.6	393	67.8	111	70.7	481	48.4
Headache frequency								
Infrequent	32	15.2	151	26.1	28	17.8	–	–
Episodic	137	72.7	367	63.4	103	65.6	–	–
Chronic	22	11.5	61	10.5	26	16.6	–	–
Age							
<16	18	9.3	24	4.1	31	19.7	154	15.5
16	55	28.5	151	26.0	50	31.8	273	27.5
17	73	37.8	221	38.1	42	26.8	345	34.7
18	47	24.4	184	31.7	34	21.7	221	22.3
Type of School							
High school	52	26.9	229	39.5	44	28.0	293	29.5
Profession oriented school	128	66.3	307	52.9	78	49.7	541	54.4
Apprentices	13	6.7	44	7.6	35	22.3	159	16.0
Smoking							
Yes	52	26.9	168	29.0	56	35.7	253	25.6
No	141	73.1	412	71.0	101	64.3	737	74.4
Alcohol consumption								
Yes	154	83.2	479	86.0	109	71.2	729	76.7
No	31	16.8	78	14.0	44	28.8	222	23.3
Socio-economic Status								
Low	3	0.6	6	1.0	1	0.6	8	0.8
Medium	65	34.2	180	31.4	55	35.5	312	32.1
High	122	64.2	388	67.6	99	63.9	652	67.1
Sleep Disturbances								
Yes	92	48.7	230	39.9	75	49.0	320	32.8
No	97	51.3	347	60.1	78	51.0	655	67.2
Screen Time								
Less/equal than 1 hour/day	13	6.9	39	6.8	12	7.7	62	6.4
Between 1 and 3 hours/day	36	19.0	147	25.8	32	20.5	252	26.1
Between 3 and 5 hours/day	76	40.2	224	39.9	44	28.2	387	40.0
Between 5 and 7 hours/day	40	21.2	85	14.9	34	21.8	150	15.5
More than 7 hours/day	24	12.9	75	13.2	34	21.8	116	12.0
Regular exercise								
Yes	103	53.9	308	53.4	81	51.9	356	36.3
No	88	46.1	269	46.6	75	48.1	624	63.7
My parents understand me								
Never	13	6.7	4	0.7	8	5.1	63	6.3
Sometimes	49	25.4	174	30.0	55	35.0	289	29.1
Almost always	131	67.9	398	68.6	94	59.9	631	63.5
Health Self-Assessment								
Poor	0	0.0	1	0.2	0	0.0	2	0.2
Fair	18	10.4	75	13.9	25	16.9	53	5.8
Good	113	65.3	341	63.4	88	59.5	519	56.4
Excellent	42	24.3	121	22.5	35	23.6	347	37.3
BMI								
<18.5	16	8.3	52	89.0	16	9.8	103	10.4
18.5–24.9	147	76.2	409	70.6	112	71.8	723	73.2
25–29.9	18	9.3	97	16.8	24	15.4	129	13.1
≥30	12	6.2	21	3.6	4	2.6	33	3.3
Mode of birth								
spontaneous	113	79.0	341	78.0	77	71.3	507	74.8
vacuum extraction	6	4.2	20	4.6	7	6.5	38	5.6
caesarean	24	16.8	76	17.4	24	22.2	133	19.6
Breastfeeding								
Yes	76	74.5	222	76.8	66	74.2	391	77.9
No	26	25.5	67	23.2	23	25.8	111	22.1
5′ APGAR-Score								
≥ 6 points	144	100.0	442	100.0	104	98.1	687	99.9
<6 points	0	0.0	0	0.0	2	1.9	1	0.1
Total	193	100.0	580	100.0	157	100.0	993	100.0

The sample size may be different per variable or not add up to total per column because of missing data. Numbers may not add to 100% due to rounding of values. TTH: Tension-Type-Headache; Headache frequency: infrequent: <1 headache day/month, episodic headache: >1 and <15 headache days/month, chronic: >15 headache days/month; Socio-Economic Status according to the Family Affluence Scale III (max. 9 points): low (0–3), medium (4–6), high (7–9); regular exercise: at least 3 days/week with 60min of physical activity; Health Self-Assessment Scale according to the Health Behaviour in School-aged Children (HBSC); BMI: Body Mass Index (kg/m2); APGAR: Appearance, Pulse, Grimace, Activity and Respiration.

### Sociodemographic data

Results of the multinomial logistic regression are shown in Table 2. Female sex and sleep disturbances were associated with a significantly higher risk for each of the headache categories (p < 0.05).

**Table 2.** Association between headache type and probable risk factors.

	Migraine			TTH			Other headache		
	(n = 193)			(n = 580)			(n = 157)		
	OR	CI (95%)	Sig.	OR	CI (95%)	Sig.	OR	CI (95%)	Sig.
Sex	
Female	1.0	1.0	1.0	1.0	1.0	1.0	1.0	1.0	1.0
Male	0.62	(0.44–0.88)	**0.007**	0.51	(0.40–0.64)	**0.001**	0.40	(0.27–0.59)	**0.001**
Age	1.08	(0.91–1.28)	0.385	1.41	(1.25–1.60)	**0.001**	0.97	(0.81–1.16)	0.752
Screen time	1.05	(0.98–1.13)	0.185	1.02	(0.97–1.07)	0.442	1.12	(1.04–1.22)	**0.005**
Physical activity	0.94	(0.86–1.04)	0.252	0.88	(0.83–0.95)	**0.001**	0.93	(0.83–1.03)	0.163
School type									
Apprentices	1.0	1.0	1.0	1.0	1.0	1.0	1.0	1.0	1.0
Highschool	1.93	(0.97–3.83)	0.062	2.23	(1.48–3.38)	**0.001**	0.70	(0.41–1.21)	0.200
Profession-oriented School	2.67	(1.41–5.10)	**0.003**	1.66	(1.12–2.46)	**0.012**	0.72	(0.44–1.16)	0.174
BMI	1.01	(0.97–1.06)	0.657	1.02	(0.99–1.06)	0.175	0.98	(0.94–1.03)	0.508
Sleep disturbance	1.92	(1.38–2.67)	**0.001**	1.32	(1.05–1.66)	**0.020**	1.70	(1.19–2.44)	**0.004**
Alcohol consumption	1.39	(0.89–2.18)	0.148	1.48	(1.09–2.02)	**0.013**	0.67	(0.44–1.02)	0.062
Smoking	0.99	(0.68–1.45)	0.968	1.08	(0.83–1.39)	0.573	1.39	(0.93–2.08)	0.112

Bold font indicates statistical significance (p < 0.05).Results for multinomial logistic regression models with the headache type as dependent variable and the no headache category as reference group. TTH: Tension-type-Headache; OR: odds ratio; CI: confidence interval; BMI: Body Mass Index (kg/m^2^); Physical activity: days/week with at least 60min of physical exercise.

High Screen time levels were associated with other headaches (OR 1.12; p < 0.01), but not with migraine or TTH. Regular exercise significantly reduced the risk for having TTH (OR 0.88; p < 0.01), whereas alcohol consumption (OR 1.48; p < 0.05) and age (OR 1.41; p < 0.05) increased the risk. Adolescents who attended a profession-oriented school were more likely to have TTH (OR 1.66; p < 0.05) or migraine (OR 2.67; p < 0.01), and adolescents from high schools were more likely to have TTH (OR 2.23; p < 0.01) compared to apprentices.

**Figure 2.**
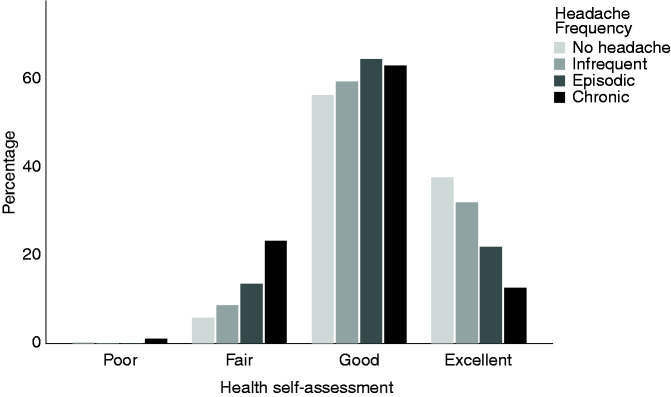
Health Self-Assessment depending on the headache frequency.

There was no association between the risk of headaches and the mode of birth, low APGAR score (defined as 5-minute APGAR Score below 6 points according to the American Association of Pediatrics (21)) or breastfeeding. Positive family history regarding hypertension and/or stroke did not influence the risk of having a primary headache disorder. The outcome of the Health-Self-Assessment is shown in Figure 2. Only 1 participant (1.9%) with chronic headaches reported poor health, whereas 64 (63.0%) and 6 (11.0%) of those within the highest headache frequency category rated their health as good and excellent, respectively.

### Sex-specific differences

Girls were more likely to suffer from headaches – the female to male ratio in the study was 2:1. The prevalence of a primary headache disorder in girls was 56.4% compared to boys 37.6% (p < 0.001). Migraine, TTH and other headache was described in 10.6%, 35.7% and 10.1% of the girls and in 9.3%, 22.8% and 5.6% of the boys, respectively. In the subgroups TTH and other headache, the prevalence was significantly higher for girls than for boys. However, there was no sex-specific difference in the migraine group.

### Use of on-demand medication

Of the 930 adolescents who reported having headache, data from 583 participants on the use of on-demand medication was available. 280 (48.2%) reported taking acute medication, almost exclusively NSAIDs (278; 47.6%) were used by the adolescents. The majority, 255 (90.7%) took a single agent preparation, 23 (8.2%) took NSAIDs in combined formulations, 2 (0.7%) used triptanes as pain relief and none took opiates or ergotamines. Data showed that girls were more likely to take medication than the male participants, although the difference was not significant (52.1% vs. 40.9%, p > 0.05). Overall, 20 (3.4%) participants were diagnosed with a medication overuse. This number increased up to 21.7% considering only participants with chronic headaches. Girls were more likely to suffer from medication overuse (4.4% vs. 1.4%, OR 3.31, p < 0.05). Importantly, the use of prophylactic medication has not been reported by any of the participants.

## Discussion

Herein, we present a large scale, community-based cross-sectional study in 14- to- 19-year-old adolescents, where information on headache characteristics and medication was collected in a structured face-to-face interview by headache specialists. Our results demonstrated that almost half of the students reported having headache. The overall prevalence of migraine and TTH headache was comparable to previous published data. (3,4,22).

From puberty onwards, girls are more likely to be affected by primary headache disorders than boys (2). In our study, sex differences were observed regarding the prevalence of the two tension-type headache and other headache subgroups. However, no significant difference could be detected in the migraine subgroup. Referring to this, our results differ from previous studies, where females were significantly more likely than males to have migraine (2,3,22). This could be related to the heterogeneous age distribution in our study and the missing data of the onset of the menarche, which is related to the beginning of migraine in girls. However, maybe this highlights the fact that migraine should be diagnosed in a structured face-to-face interview, as we did, and not by only using questionnaires as utilized in many studies before (3,8,22).

Headache during adolescence seems to be related to life-style factors – irregular sleeping patterns, age, physical inactivity, alcohol consumption, high BMI, high screen time exposure and smoking pose a potential risk for having headaches (7,8,23–25).

In the present sample, sleeping disorders were associated with a higher risk for migraine, TTH and other headache. Sleep disturbance can either be seen as a trigger or as cause for the headaches and might contribute to their chronification (8). Therefore, attention should be paid to adequate sleep hygiene, especially in adolescents with headaches. Our results suggest that there is an association between low- to moderate alcohol consumption and high level of physical activity with reduced risk of TTH. It is difficult to determine whether headaches are the reason for the inactivity or the other way round as our data was collected only observatory. Therefore, our data cannot prove that physical activity is effective as therapeutic intervention in adolescents. Still, we argue that our data corroborate the importance of physical activity as data from adults show (26). We calculated that 5 days with 60 minutes of exercise per week could reduce the risk of TTH by half. To the best of our knowledge, this is the first study to show such a profound influence of regular physical exercise on TTH in adolescents.

High cumulative screen time was independently related to a higher risk of other headache but not migraine or TTH in our cohort. This is somehow surprising since a recent published study involving 4927 young adults found an association between high screen time exposure and migraine, primarily with aura, but not for non-migraine headache. As we did not differentiate between migraine with and without aura, the interpretation of our data, concerning a missing correlation of screen time exposure with migraine, must be taken carefully.

NSAIDs, primarily single agent preparations, were the most widely used on-demand medication. Most importantly only two adolescents in the cohort treated their migraine headaches with triptans, although the use of Sumatriptan and Zolmitriptan has been approved in Austria for over 12-year-olds. However, at the time of study conduct these pharmaceuticals were not available as over-the-counter (OTC) and the first prescription had to be done by a neurologist, which none of the participants has consulted for their headaches.

The prevalence of medication overuse among adolescents with chronic headache was 21.7%. Girls were three times more likely to be affected than boys. Our data is in line with previous studies – the prevalence of medication overuse in children and adolescents is estimated at 20–60% (27,28). Although we did not directly evaluate Medication Overuse Headache (MOH), it is likely that some adolescents meet the criteria. Piazza et al. (29) reported a prevalence of 20.8% MOH in children and adolescents with chronic headache and Wang et al. (30) described a prevalence of 20.0%. Most adolescents self-medicate with OTC medications, mainly without consulting their parents or having the knowledge about the effects and possible side effects of the drugs (31).

Despite the high headache frequency in the study population, none used prophylactic medications or underwent behavioural therapy. Our data confirm the unmet medical need for a sufficient medical care and treatment of juvenile headache patients. This unmet need is sadly reflected by the fact that headache outpatient clinics, specialized in paediatric headache are scarce all over Europe.

Chronic headaches have an enormous impact on the students’ performance at school, social life, and leisure time. Migraine and TTH are estimated to account for 37.5% of all-cause prevalence and for 7% of all-cause years lived with disability (YLDs) in the global burden of disease (GBD) study in children and adolescents aged from 5 to 19 years (32).

Although headaches are known to have a significant impact on the quality of life (32,33), there seems to be a discrepancy in some participant’s health perception, as shown in Figure 2. In one questionnaire the teenagers had to rate their health as either “poor”, “fair”, “good” or “excellent”. Despite having chronic headaches, 63% described their own health as good and 11% even rated their health as excellent. To the best of our knowledge, this is the first study to report a mismatch between the perception of one’s own health and the frequency of headaches, suggesting that headaches are trivialized in adolescence and not seen as “relevant disorder” or health issue.

Our results showed a decrease in the prevalence of all headache types at the age of 18, predominantly in migraines. We are fully aware of the fact that this seems contradictory to literature and clinical experience. However, we would like to point out that migraine prevalence in this age group varies widely in the literature between 3 -15% (1), and therefore our results are still within the published range. If this observation is due to the lower number of adolescents or can simply be explained by the variance in the respective age groups remains speculative.

### Strengths and Limitations

A strength of the present study is the large number of participants. It was conducted at 45 different sites in North-and East-Tyrol (Austria) as well as South-Tyrol (Italy). The 2000 participants represent about 5% of the Tyrolean population at that age and hence allow in-depth insights into the headache prevalence and headache features of a representative Middle European population. Within the framework of the present study, many parameters were collected, thus allowing an excellent characterisation of the affected adolescents. Only well accepted scales and scores were used. All participants were examined in a standardized manner and headaches were defined according to the latest ICHD-3. The structured face-to-face headache interviews were carried out by experienced headache specialists, who were trained at our tertiary headache centre in Innsbruck, reducing misclassification bias. Thus, the collected data can be considered valid, comparable, and up to date.

Some limitations of our study must be reported. The interviews are the sole source of information on headaches. Several studies compared the data quality of retrospective structured face-to-face interviews and questionnaires with that of prospective headache diary entries (34–36). The variation in the methodology of the surveys make it difficult to compare them and might be the reason of the heterogeneity of the available data on primary headache disorders in children and adolescents. A combination of different sources of information might be the most suitable way to get a comprehensive picture of the prevalence and the characteristics of headache disorders in the young population.

## Conclusion

Despite the high prevalence of primary headache disorders in adolescents there is a lack of paediatric headache outpatient clinics, which causes an insufficient medical care for teenagers. Consequently, the correct headache diagnosis is often missed, and adequate treatment denied. Preventive non-drug and pharmacological treatments are not established in this young patient population and could explain the high number of adolescents with medication overuse in the present study. Nevertheless, the benefits of lifestyle modifications such as regular physical exercise and optimal sleeping patterns as well as cognitive behavioural therapy should be emphasized. It is important to better characterize the affected population and to identify possible risk factors to provide points of action for preventive measures.

Our data support the medical need to put the trivialization of headaches aside. It is important to promote health education already in teenagers and to encourage public awareness, including that of health care providers.

## Contributors

KK – Planned and carried out statistical analysis. Drafted and wrote the manuscript.

FF – Drafted and revised the manuscript.

BB – Carried out data extraction and statistical analysis. Revised the manuscript.

SK – Participated in the EVA-Tyrol data collection. Carried out data extraction. Revised the manuscript.

BW – Participated in the EVA-Tyrol data collection and revised the manuscript.

UK – Principal Investigator of the EVA-Tyrol-project. Planned the study. Revised the manuscript.

MK – Principal Investigator of the EVA-Tyrol-project. Planned the study. Substantially revised the manuscript.

GB – Planned the study. Drafted and wrote the manuscript.

All authors read and approved the final manuscript.

## Clinical implications

- Regular physical activity can reduce the risk for TTH by half.
- 11.9% of adolescents fulfil the diagnostic criteria for chronic headache of which 21.7% overuse acute medication.
- Adolescents perceive themselves as healthy despite suffering from chronic headaches.
- The use of prophylactic medication and that of triptans as acute medication is not well established in adolescents.

## Appendix

Additional members of the EVA Tyrol Study Group:

Carmen Reiter, Department of Paediatrics II, Medical University of Innsbruck, Innsbruck, Austria

Christina Schreiner, Department of Paediatrics II, Medical University of Innsbruck, Innsbruck, Austria

Julia Klingenschmid, Department of Paediatrics II, Medical University of Innsbruck, Innsbruck, Austria

Julia Marxer, Department of Paediatrics II, Medical University of Innsbruck, Innsbruck, Austria

Mandy Asare, Department of Neurology, Medical University of Innsbruck, Innsbruck, Austria and VASCage, Research Centre on Vascular Ageing and Stroke, Innsbruck, Austria

Manuela Bock-Bartl, VASCage, Research Centre on Vascular Ageing and Stroke, Innsbruck, Austria

Martina Kothmayer, Department of Paediatrics II, Medical University of Innsbruck, Innsbruck, Austria

Maximilian Bohl, Department of Neurology, Medical University of Innsbruck, Innsbruck, Austria

Maximilian Pircher, Department of Paediatrics II, Medical University of Innsbruck, Innsbruck, Austria

Raimund Pechlaner, Department of Neurology, Medical University of Innsbruck, Innsbruck, Austria

Nina Gande, Department of Paediatrics II, Medical University of Innsbruck, Innsbruck, Austria

Anna Staudt, Department of Paediatrics II, Medical University of Innsbruck, Innsbruck, Austria

Katharina Stock, Department of Paediatrics III, Medical University of Innsbruck, Innsbruck, Austria

Christoph Hochmayr, Department of Paediatrics II, Medical University of Innsbruck, Innsbruck, Austria

Ralf Geiger, Department of Paediatrics III, Medical University of Innsbruck, Innsbruck, Austria

Stefan Kiechl, Department of Neurology, Medical University of Innsbruck, Innsbruck, Austria and VASCage, Research Centre on Vascular Ageing and Stroke, Innsbruck, Austria
